# Supplementary material for: OPERA-LG: efficient and exact scaffolding of large, repeat-rich eukaryotic genomes with performance guarantees
Source: Genome Biol. 2016 May 11;17:102. doi: 10.1186/s13059-016-0951-y (PMC4864936; doi:10.1186/s13059-016-0951-y)
Supplement: Additional file 1: — Contains supplementary notes, figures and tables. (DOCX 2996 kb) [file 13059_2016_951_MOESM1_ESM.docx]

**OPERA-LG: Efficient and exact scaffolding of large, repeat-rich eukaryotic genomes with performance guarantees**

Song Gao, Denis Bertrand, Burton KH Chia, Niranjan Nagarajan

**Supplementary Note 1: Correcting for polyploidy and aneuploidy**

Most assembly programs are designed with assumptions specific to haploid genomes and correspondingly applying them to non-haploid genomes can lead to unexpected results and greater fragmentation of assembly than is typically induced by repetitive sequences. Also, for aneuploid genomes (as is frequently seen in cancer tissues and cell lines), the straightforward approach described previously to identify unique contigs may be too conservative in identifying non-repeat sequences (where repeats are defined as being sequences with multiple distinct locations in the genome).

For assembling polyploid or aneuploid genomes, OPERA-LG uses the haploid coverage of the genome ($H$, user-specified) to estimate the copy number for each contig as $max(1, round\left( \frac{C}{H} \right))$ (where $C$ is the average coverage of the contig). With sufficient coverage and single-copy contigs, more sophisticated, model-fitting-based methods can be used to directly estimate haploid genome coverage from coverage statistics. Based on the copy number of contigs, OPERA-LG clusters and scaffolds contigs with the same copy number (removing edges between contigs whose average coverage differs $>H/2$), assuming that all contigs with copy number less than the maximum ploidy (user-specified, but can be obtained from coverage statistics) are classified as unique. While this assumption can lead to scaffold errors when, for example, a two-copy repeat from a haploid region is linked by a scaffold edge to a unique contig from the diploid genome, such cases are likely be relatively infrequent. In balance, this assumption allows OPERA-LG to correctly scaffold unique contigs (and repeat contigs) from polyploid chromosomes, which can form a significant fraction of the genome to be assembled. Furthermore, estimation of copy number for scaffold edges can enable even more reliable scaffolding of polyploidy genomes. However, this would require development of improved models that account for mapping biases and is beyond the scope of this work. For diploid genomes, the --hybrid-scaffold option in OPERA-LG allows haploid and diploid contigs to be scaffolded together.

**Supplementary Note 2: Alternative inference of scaffold edges from long reads**

In addition to the approach used in SSPACE-LR to construct scaffold edges, we also assessed an alternative ad hoc approach that minimizes changes to the OPERA-LG framework. Specifically, we aligned PacBio reads to contigs using BLASR [26] with default parameters. Reads aligned to multiple contigs were then used to construct synthetic mate-pairs connecting every pair of contigs aligned to the read. Only alignments containing more than 90% of the contig or where read and contig ends overlapped were considered for this analysis. Synthetic mate-pairs with estimated distances in the range [0-300], [300-1,000], [1,000-2,000], [2,000-5,000], [5,000-15,000] and [15,000-40,000] were then provided as synthetic libraries for OPERA-LG to construct scaffold edges and scaffold with.

(a) (b)

(c)

**Supplementary Figure 1**: **Key algorithmic steps in OPERA-LG.** a) Memoized Search: the search procedure in OPERA-LG is akin to a depth-first search where previously visited partial scaffolds (the tail of which is defined by a list of contigs i.e. “Active Region” or AR and a set of incident edges i.e. “Dangling Edges” or DE) are “memoized” (as <AR, DE> pairs defining an equivalence class of partial scaffolds and not re-searched). b) Graph Contraction: the subgraph demarcated by dotted lines is independently solved in OPERA-LG, allowing for significant runtime improvements. Border contigs are large contigs (longer than the library size upper bound) such that no concordant scaffold edges can span them. c) Gap-size Optimization: gap sizes are jointly optimized in OPERA-LG by minimizing the quadratic function depicted in the figure.


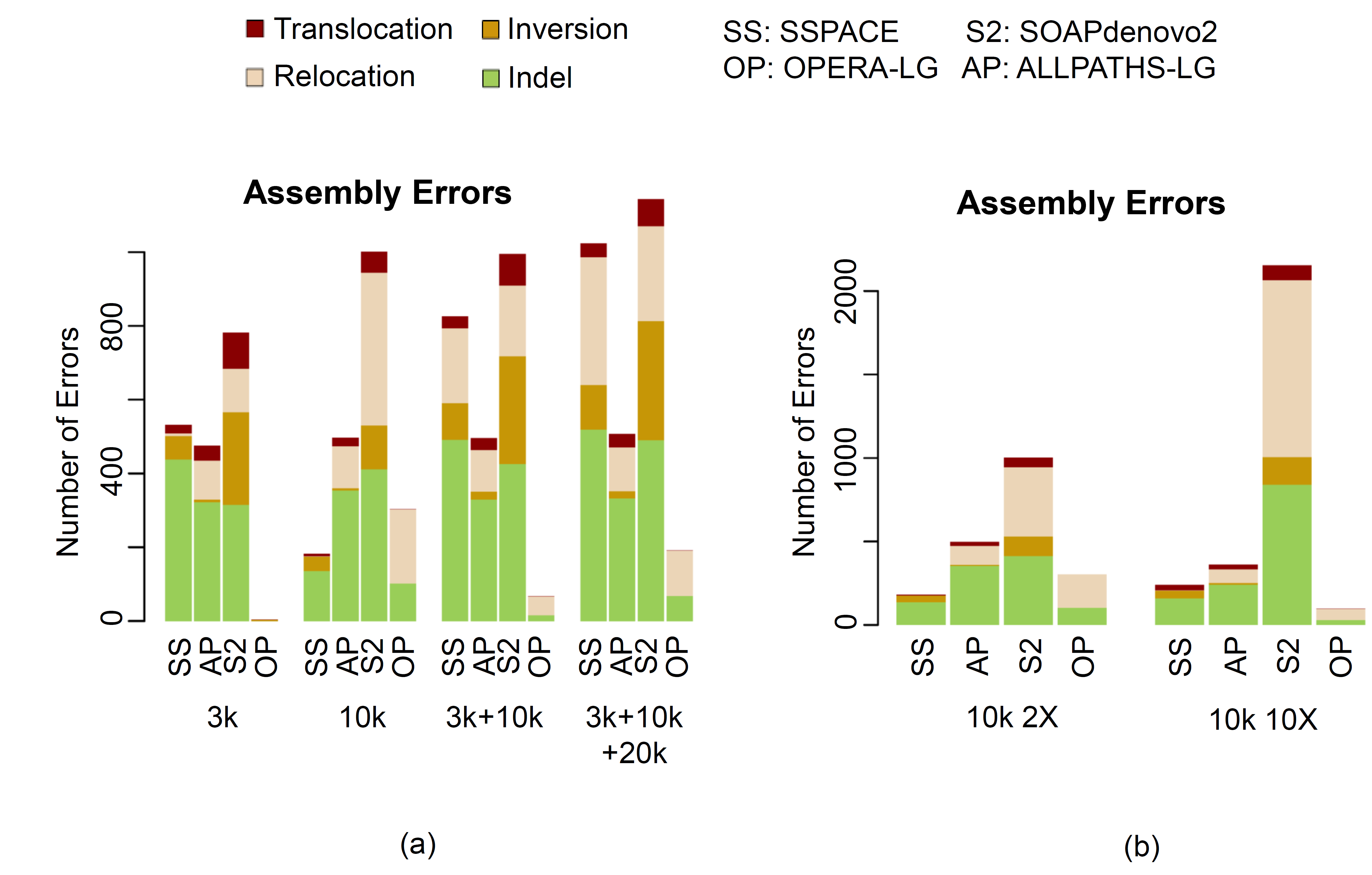

 (a) (b)

**Supplementary Figure 2**: **Assembly performance as a function of library information and sequencing depth.** (a) Assembly errors as a function of the mate-pair libraries that were provided as input. (b) Assembly errors as a function of sequencing depth. Results shown here are for the *C. elegans* dataset.

(a) (b)

**Supplementary Figure 3**: **Assembly performance as a function of library quality.** Results shown are for the *D. melanogaster* dataset using 10 kbp libraries.

**
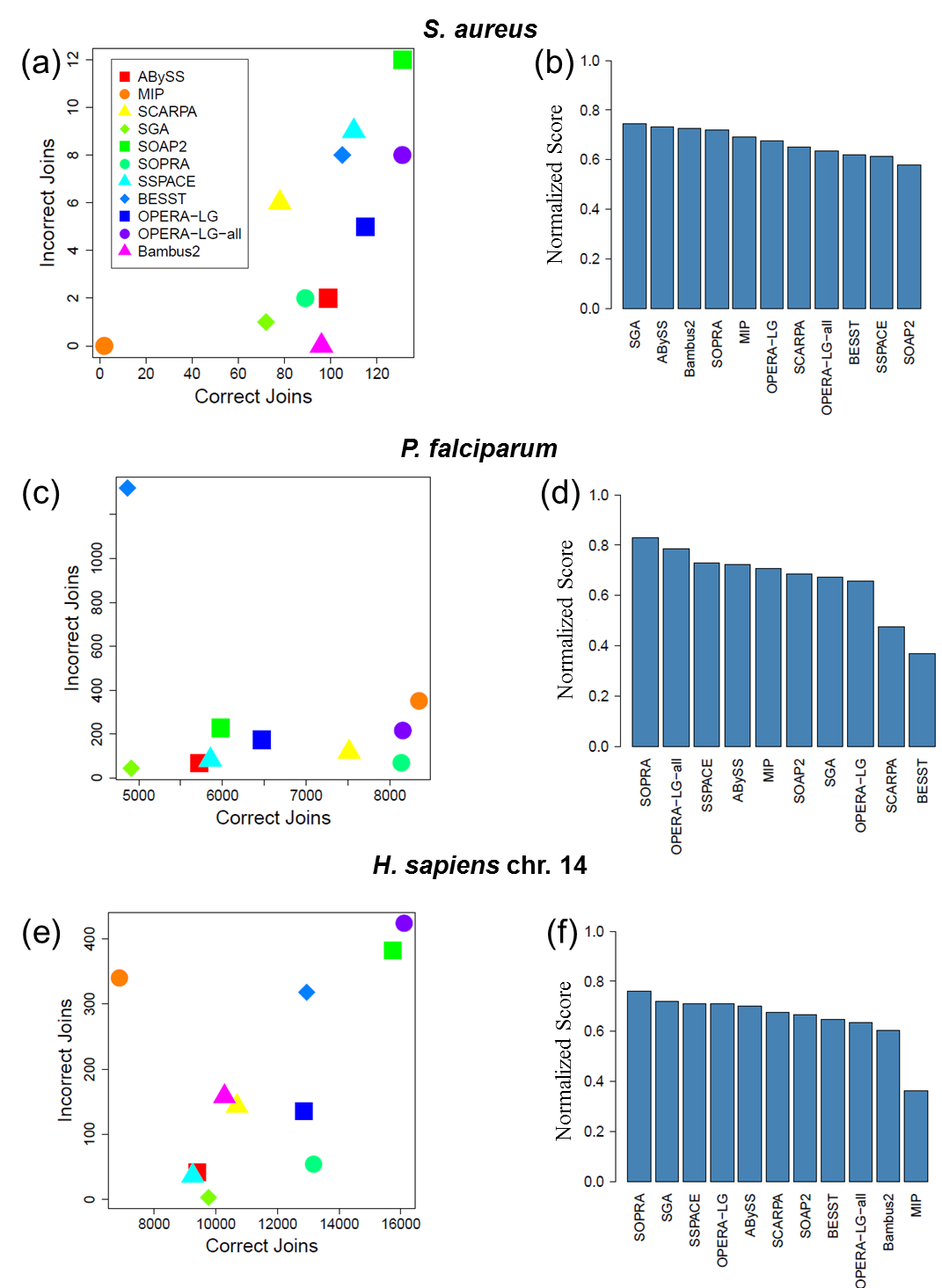
Supplementary Figure 4**: **Evaluation using an alternative metric of scaffold quality.** (a), (b) *S. aureus* results (c), (d) *P. falciparum results* (e), (f) *H. sapiens* chromosome 14 results. The “normalized score” integrates the number of incorrect joins, correct joins and missing contigs into a single *ad hoc* weighted score. OPERA-LG-all results were obtained by running OPERA-LG on the full contig set (including contigs smaller than 500bp).

**
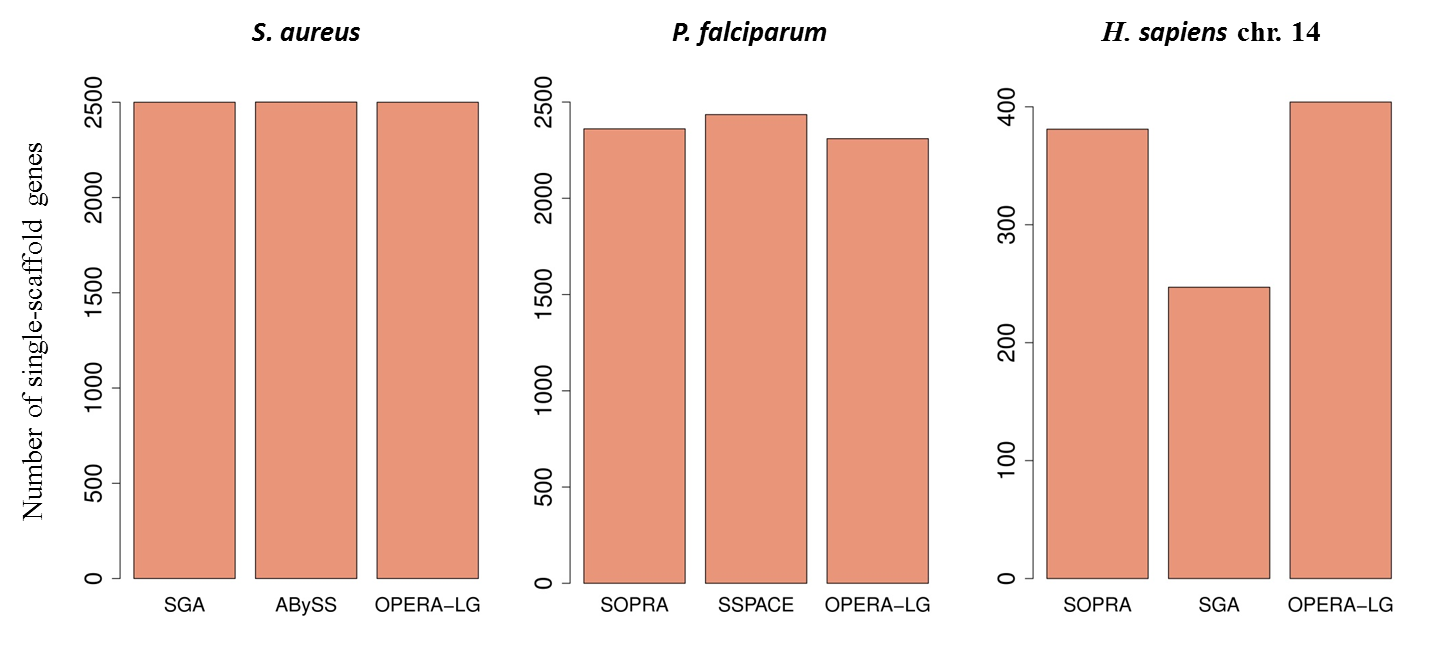
Supplementary Figure 5**: **Evaluation of assemblies in terms of single-scaffold genes.** To evaluate the ability of assemblies to accurate capture genes without fragmenting or having missing sequences, we computed the number of genes that aligned to a single scaffold in the assembly with coverage ≥ 0.95 and average identity ≥ 0.95 (using BLAST with E-value threshold of 10^-5^). For each dataset from Hunt et al. we compared OPERA-LG to the two best assemblies in terms of the normalized score. As shown here, OPERA-LG is comparable to other assemblies in terms of the number of genes captured (independent of normalized scores), while providing a significant improvement in terms of corrected N50 for the *H. sapiens* dataset (**Figure 4d**).

(a) (b)

**Supplementary Figure 6**: **Observed and un-observed read-pairs.** (a) Graphical depiction of the phenomena of mate-pairs connecting contigs coming from a truncated distribution defined by contig lengths (c_A_ and c_B_) and gap size (g). (b) Empirical distribution of the distance between observed mate-pairs (mean μ and standard deviation $\sigma$), and the region of truncation (defined by $g$ and $C$).

| ScaffoldWithRepeat($S', p$)  **Require:** A scaffold graph $G=(V, E)$ and a partial scaffold $S'$ with at most $p$ discordant edges.  **Ensure:** Return a scaffold $S$ of $G$ with at most $p$ discordant edges and where $S'$ is a prefix of $S$  1: **if** $S'$ is a scaffold of $G$, **then** 2: return $S'$ 3: **end if** 4: **for** every $c\in V-V_{S'}$ in each orientation **do** 5: Let $S''$ be the scaffold formed by concatenating $S'$ and $c$; 6: **If** a confirmed repeat $r$ should be removed **then** 7: trace back to the contig before $r$; 8: **else** 9: Let $A$ be the active region of $S''$; 10: Let $D$ be the set of dangling edges of $S''$; 11: Let $k$ be the number of discordant edges in $S''$; 12: **if** $(A,D,k)$ is unmarked, **then** 13: Mark $(A,D,k)$ as processed; 14: **if** $k\leq p$, **then** 15: $S'''\leftarrow$ ScaffoldWithRepeat($S'', p$); 16: **if** $S'''\neq$ FAILURE, return $S'''$; 17: **end if** 18: **end if** 19: **end if** 20: **end for** 21: Return FAILURE; |
| --- |

**Supplementary Figure 7**: **An algorithm for generating a minimal-repeat optimal scaffold with at most** $\boldsymbol{p}$ **discordant edges.**

**Supplementary Figure 8**: **An example of the prefix tree data structure used to record visited partial scaffolds.** The example here records the partial scaffolds S_1_, S_2_ and S_3_ shown at the top of the figure, where A_i_ and X_i_ represent the active region (list of contigs in the tail of the scaffold) and discordant edges, respectively, of the partial scaffolds.

|  | | **Contigs** | **Scaffolds** |
| --- | --- | --- | --- |
| *D. melanogaster* | SSPACE | n.a. | 92 |
|  | SOAPdenovo2 | 86 | 95 |
|  | SOPRA | n.a. | 96 |
|  | BESST | n.a. | 91 |
|  | OPERA-LG | n.a. | 92 |
|  | ALLPATHS-LG | 89 | 94 |
| *C. elegans* | SSPACE | n.a. | 107 |
|  | SOAPdenovo2 | 86 | 102 |
|  | SOPRA | n.a. |  |
|  | BESST | n.a. | 99 |
|  | OPERA-LG | n.a. | 100 |
|  | ALLPATHS-LG | 94 | 100 |
| *H. sapiens* | SSPACE | n.a. | 112 |
|  | SOAPdenovo2 | 66 | 94 |
|  | SOPRA | n.a. |  |
|  | BESST | n.a. | 85 |
|  | OPERA-LG | n.a. | 106 |
|  | ALLPATHS-LG | 79 | 93 |

**Supplementary Table 1. Assembly size for results reported in Figure 3a-d.** The numbers presented here show the total length of contigs and scaffolds (longer than 500 bp) in each assembly, reported as a percentage of genome length. Note that scaffold lengths include gaps and can exceed 100% due to the use of a lower bound for gap sizes in many scaffolders. SOPRA did not finish scaffolding for the *C. elegans* and *H. sapiens* datasets after 10 days and was stopped.

| **Scaffold Contiguity** | | | | | | |
| --- | --- | --- | --- | --- | --- | --- |
|  | | | **N50 (Mbp)** | | **Corrected N50 (Mbp)** | |
| *D. melanogaster* | SSPACE | | 1.71 | | 0.84 | |
|  | SOAPdenovo2 | | 12.52 | | 0.82 | |
|  | SOPRA | | 0.40 | | 0.30 | |
|  | BESST | | 7.12 | | 0.94 | |
|  | OPERA-LG | | 12.00 | | 8.79 | |
| *C. elegans* | SSPACE | | 0.32 | | 0.15 | |
|  | SOAPdenovo2 | | 4.41 | | 0.11 | |
|  | SOPRA | |  | |  | |
|  | BESST | | 0.70 | | 0.09 | |
|  | OPERA-LG | | 8.01 | | 2.60 | |
| *H. sapiens* | SSPACE | | 0.21 | | 0.10 | |
|  | SOAPdenovo2 | | 1.53 | | 0.11 | |
|  | SOPRA | |  | |  | |
|  | BESST | | 0.36 | | 0.27 | |
|  | OPERA-LG | | 5.20 | | 1.46 | |
| **Scaffold Errors** | | | | | | |
|  | | | **Indel** | **Inversion** | **Relocation** | **Translocation** |
| *D. melanogaster* | | SSPACE | 376 | 33 | 87 | 40 |
|  |  | SOAPdenovo2 | 196 | 106 | 104 | 30 |
|  |  | SOPRA | 222 | 144 | 309 | 144 |
|  |  | BESST | 100 | 282 | 71 | 186 |
|  |  | OPERA-LG | 30 | 0 | 29 | 1 |
| *C. elegans* | | SSPACE | 854 | 126 | 630 | 39 |
|  |  | SOAPdenovo2 | 493 | 323 | 253 | 71 |
|  |  | SOPRA |  |  |  |  |
|  |  | BESST | 341 | 921 | 248 | 458 |
|  |  | OPERA-LG | 67 | 0 | 123 | 0 |
| *H. sapiens* | | SSPACE | 9,600 | 1,209 | 8,504 | 676 |
|  |  | SOAPdenovo2 | 26,408 | 1,033 | 24,442 | 1,430 |
|  |  | SOPRA |  |  |  |  |
|  |  | BESST | 2,127 | 774 | 751 | 562 |
|  |  | OPERA-LG | 7,297 | 60 | 4,758 | 94 |

**Supplementary Table 2. Scaffold contiguity and errors on synthetic datasets.** All programs were provided with the same set of contigs (generated by SOAPdenovo) and the final assemblies were only corrected for scaffold errors. A subset of these results are depicted in **Figure 3a**, **b**. SOPRA did not finish scaffolding for the *C. elegans* and *H. sapiens* datasets after 10 days and was stopped.

|  | | **Indel** | **Inversion** | **Relocation** | **Translocation** |
| --- | --- | --- | --- | --- | --- |
| *D. melanogaster* | ALLPATHS-LG | 208 | 38 | 84 | 49 |
|  | SOAPdenovo2 | 194 | 107 | 101 | 31 |
|  | OPERA-LG | 34 | 0 | 29 | 1 |
| *C. elegans* | ALLPATHS-LG | 332 | 19 | 119 | 37 |
|  | SOAPdenovo2 | 490 | 322 | 258 | 73 |
|  | OPERA-LG | 67 | 0 | 123 | 1 |
| *H. sapiens* | ALLPATHS-LG | 18,614 | 240 | 5,834 | 3,082 |
|  | SOAPdenovo2 | 26,567 | 1,227 | 24,443 | 1,781 |
|  | OPERA-LG | 7,310 | 63 | 4,761 | 161 |

**Supplementary Table 3. Number of assembly errors as depicted in Figure 3d.** These results include contig and scaffold errors.

|  | **N50 (Mbp)** | **Corrected N50 (Mbp)** | **Number of Errors** |
| --- | --- | --- | --- |
| **SSPACE** | 1.4 | 0.3 | 555 |
| **SOAPdenovo2** | 7.0 | 0.8 | 574 |
| **ALLPATHS-LG** | 12.0 | 1.1 | 432 |
| **OPERA-LG** | 12.0 | 12.0 | 14 |

**Supplementary Table 4. Impact of long reads on assembly results.** The results reported here are based on redoing the analysis reported in **Figure 3c** for *D. melanogaster*, where 250 bp reads were simulated (instead of 80 bp and genome coverage was kept the same) for the paired-end read library (fragment size 400 bp).

| **Scaffold Evaluation** | | | | | | | | | |
| --- | --- | --- | --- | --- | --- | --- | --- | --- | --- |
|  | | | **N50 (Mbp)** | | | **Corrected N50 (kbp)** | | | **Number of Errors** |
| *C. sinensis* | SSPACE | | 0.26 | | | 0.05 | | | 5,020 |
|  | SOAPdenovo2 | | 0.26 | | | 0.07 | | | 5,455 |
|  | SOPRA | |  | | |  | | |  |
|  | BESST | | 0.06 | | | 0.04 | | | 1,827 |
|  | OPERA-LG | | 0.46 | | | 0.18 | | | 1,492 |
| *P. stipitis* | SSPACE | | 0.31 | | | 0.09 | | | 74 |
|  | SOAPdenovo2 | | 0.40 | | | 0.30 | | | 61 |
|  | SOPRA | | 0.23 | | | 0.22 | | | 8 |
|  | BESST | | 0.10 | | | 0.09 | | | 104 |
|  | OPERA-LG | | 0.32 | | | 0.32 | | | 1 |
| **Scaffold Errors** | | | | | | | | | |
|  | | | | **Indel** | **Inversion** | | **Relocation** | **Translocation** | |
| *P. stipitis* | | SSPACE | | 7 | 6 | | 46 | 15 | |
|  |  | SOAPdenovo2 | | 19 | 6 | | 20 | 16 | |
|  |  | SOPRA | | 2 | 1 | | 3 | 2 | |
|  |  | BESST | | 28 | 10 | | 48 | 18 | |
|  |  | OPERA-LG | | 0 | 0 | | 0 | 1 | |

**Supplementary Table 5. Scaffold evaluation on real datasets.** Errors for *C. sinensis* and *P. stipitis* were computed using the REAPR pipeline (no gold-standard reference) and the GAGE pipeline (gold-standard reference; scaffold errors only) respectively. Note that the REAPR pipeline does not provide a detailed breakdown of scaffold error types.

| **Scaffold Evaluation** (Synthetic Reads) | | | | |
| --- | --- | --- | --- | --- |
|  | | **N50 (kbp)** | **Corrected N50 (kbp)** | **Number of Errors** |
| *D. melanogaster* (PacBio, 7X) | LINKS | 62.1 | 62.1 | 0 |
|  | SSPACE-LR | 272.5 | 248.2 | 192 |
|  | OPERA-LG | 242.8 | 242.6 | 41 |
| *C. elegans* (PacBio, 7X) | LINKS | 9.6 | 9.6 | 0 |
|  | SSPACE-LR | 99.2 | 87.7 | 200 |
|  | OPERA-LG | 108.9 | 107.5 | 58 |
| **Scaffold Evaluation** (Real data) | | | | |
|  | | **N50 (kbp)** | **Corrected N50 (kbp)** | **Number of Errors** |
| *S. cerevisiae* (ONT, 100X) | LINKS | 193 | 137 | 10 |
|  | SSPACE-LR | 333 | 136 | 60 |
|  | OPERA-LG | 249 | 158 | 46 |
| *D. melanogaster* (PacBio, 30X) | LINKS | 190 | 183 | 416 |
|  | SSPACE-LR | 7,982 | 536 | 568 |
|  | OPERA-LG | 1,761 | 1,290 | 498 |
| *M. undulatus* (PacBio, 7X) | LINKS |  |  |  |
|  | SSPACE-LR |  |  |  |
|  | OPERA-LG | 12.5 | 11.1 | 26,162 |
|  | OPERA-LG-A | 31.4 | 26.2 | 17,873 |

**Supplementary Table 6. Evaluation of long read scaffolding methods.** Errors for *M. undulatus* were computed using the REAPR pipeline (5kbp library). For all other data sets, errors were computed using the GAGE pipeline (scaffold errors only). On the *M. undulatus* dataset, LINKS required >500Gb memory while SSPACE-LR did not complete after 10 days, and hence neither of the methods could be evaluated. SSPACE-LR’s scaffold edges were also used for OPERA-LG analysis by default. OPERA-LG-A refers to results from an alternate approach to generate scaffold edges as described in **Supplementary Note 2**. Values in parentheses indicate read sequencing platform and genome coverage. All methods were provided the same set of contigs: SOAPdenovo assembly of synthetic or real reads for *D. melanogaster, C. elegans*, and *M. undulates*; and for *S. cerevisiae* we used the short read assembly provided by [http://schatzlab.cshl.edu/data/nanocorr/W303_Miseq_Assembly.fa.gz](http://labshare.cshl.edu/shares/schatzlab/www-data/nanocorr/2015.07.07/W303_Miseq_Assembly.fa.gz).
